# hnRNPA2B1 regulates the alternative splicing of BIRC5 to promote gastric cancer progression

**DOI:** 10.1186/s12935-021-01968-y

**Published:** 2021-05-27

**Authors:** Wei-zhao Peng, Jin Zhao, Xin Liu, Chao-feng Li, Shuang Si, Ren Ma

**Affiliations:** Department of General Surgery, China-Japan Friendship Hospital, Beijing, 100029 China

**Keywords:** hnRNPA2B1, BIRC5, Splicing, Gastric cancer

## Abstract

**Background:**

Systematic profiling studies have implicated regulators of pre-mRNA splicing as important disease determinants in gastric cancer (GC), but the underlying mechanisms have remained elusive. Here we focused on hnRNPA2B1 splicing factor-dependent mechanisms governing GC development.

**Methods:**

The expression of hnRNPA2B1 was analyzed among the Cancer Genome Atlas (TCGA) datasets of GC and validated at mRNA level. The function of hnRNPA2B1 in GC cells was analyzed and its downstream gene was identified using RNA immunoprecipitation. Further, effect of hnRNPA2B1 on BIRC5 alternative splicing was investigated.

**Results:**

We show that overexpression of hnRNPA2B1 in GC is correlated with poor survival, and hnRNPA2B1 is required for maintaining GC malignant phenotype by promoting cell proliferation, inhibiting cell apoptosis and increasing cell metastasis. Mechanistically, hnRNPA2B1 co-expressed with several core spliceosome components and controls alternative splicing of anti-apoptotic factor BIRC5. BIRC5 isoform 202 (BIRC5-202) played the oncogenic function in GC cells, and overexpression of the BIRC5-202 transcript partly rescued the decrease in cisplatin resistance induced by downregulation of hnRNPA2B1.

**Conclusions:**

We demonstrate that hnRNPA2B1 regulates BIRC5 splicing and might act as a therapeutic target of chemo-resistant GC cells.

**Supplementary Information:**

The online version contains supplementary material available at 10.1186/s12935-021-01968-y.

## Background

It is estimated that over 27,600 cases of gastric cancers (GC) will be diagnosed in 2020, and the number of deaths will reach 11,010 [1]. China has always been severely affected by GC, accounting for about half of the GC patients in the world [2]. Statistics in 2015 found that the annual incidence of GC in China is about 679, 100, and the mortality rate ranks second among all tumors [3]. The occurrence and development of GC are affected by the interactions of complex genetic aberrations and environmental factors. Emerging studies through high-throughput sequencing and other methods have deepened our understanding of the pathogenesis of GC at the molecular level [2, 4]. Based on large-scale multi-omics sequencing research in 2014, the TCGA working group used molecular characteristics to divide GC into four categories [5]. However, the 5-year survival rate of GC patients is very low since early diagnosis of GC is challenging, and most patients are already at a late stage at the time of diagnosis [3].

For advanced patients, the efficiency of systemic chemotherapy is very limited and combined chemotherapy is more toxic. In addition, similar to other tumors, drug resistance often occurs in GC, but the biology of drug resistance is still unclear [6]. The identification of tumor cancer stem cells (CSCs) demonstrated that these cells can resist conventional therapies such as radiotherapy and chemotherapy and continue to grow after therapies, which may be the cause of recurrence after treatment [7]. CSCs are described as the distinctive subpopulation in certain tumors that retain the ability to initiate tumor proliferation and sustain both self-renewal and metastatic ability [8]. Of note, gastric CSCs were reported in 2007, although the origin of these cells were still not clear [8]. Therefore, finding critical molecules and pathways that targeting gastric CSCs is important to prevent tumor regrowth and obtain drug resistance.

As a member of the nuclear heterogeneous ribonucleoprotein (hnRNPs) family, hnRNPA2B1 is involved in transcription and RNA metabolism after binding to mRNA, including alternative splicing of pre-mRNA, RNA nuclear transport, translation, and stability regulation. hnRNPA2B1 expression is upregulated in a range of solid tumors including ovarian cancer, pancreatic ductal adenocarcinoma, hepatocellular carcinoma and pancreatic cancer [9–12]. These previous studies showed that hnRNPA2B1 promotes the malignant capability of cancer cells from several aspects, in addition, hnRNPA2B1 can regulate key molecular pathways at transcriptional, post-transcriptional or even post-translational level. A large-scale sample analysis comparing GC tissues and adjacent normal tissues uncovered that the RNA and protein levels of hnRNPA2B1 were dramatically overexpressed in GC tissues [13]. Another study showed that hnRNPA2B1 in GC cells was localized in the nuclear matrix to differentially interact with oncogenes and tumor suppressors [14]. However, whether hnRNPA2B1 can regulate GC progression by modulating CSC properties, and what is the underlying mechanism are still unclear.

In the present study, we observed that the expression hnRNPA2B1 was aberrantly upregulated in GC and hnRNPA2B1 was essential for GC cell metastasis and cisplatin resistance. The multi-omics analyses identified the inhibitor of apoptosis family member BIRC5 as the functional target of hnRNPA2B1 in GC cells. hnRNPA2B1 regulated the alternative splicing of BIRC5 mRNA and the increased BIRC5-202 expression promoted growth and metastasis in GC cells. Taken together, our results demonstrated the essential oncogenic roles of hnRNPA2B1 in GC development.

## Methods

### Cell culture

 The GC cell lines HGC-27 and MGC-803, as well as the immortalized human normal gastric epithelial cell line HFE145 used in this study were obtained from the ATCC and cultured in DMEM medium supplemented with 10 % fetal bovine serum and antibiotics (100 U/mL of penicillin and 100 mg/L of streptomycin). Cells were grown in a 5 % CO_2_ atmosphere at 37 °C. Cell lines were tested for mycoplasma contamination but have not been re-authenticated.

### Gene expression and survival analysis

The cBioPortal (cBio Cancer Genomics Portal) TCGA dataset was used to examine gene mutations with two cohorts of gastric cancer datasets: Nature 2011 [15] and OncoSG 2018 [16]. To analyze gene expression, the GC microarray and RNA-seq data were downloaded from The Cancer Genome Atlas database (http://cancergenome.nih.gov). The extraction files were imported into Partek Genomic Suite Software (Partek Inc., Chesterfield, MO, USA). Gene expression data were normalized and log2 transformed. Then principal component analysis was performed to identify outliers and artifacts on the microarray. After quality check, the one-way analysis of variance (ANOVA) model using the method of moments was applied to identify differentially-expressed genes between tumor and control group or between neoplasm histologic stages of patients with the Fisher’s least significant difference (LSD) contrast method. Kaplan-Meier Plotter database (http://kmplot.com/analysis/) [17] was used for overall survival analysis using default parameters in GEO and TCGA datasets for GC patients.

### Sample collection

 Informed consent was obtained from all individual participants included in this study. The study recruitment processes and protocol were approved by the Ethics Review Committee of China-Japan Friendship Hospital. GC tissues and paired adjacent normal tissues were obtained from patients who underwent surgery at China-Japan Friendship Hospital between May 2013 and July 2018.

### RNA isolation and qRT-PCR analysis

Total RNA was extracted from cells by using Trizol reagent (Invitrogen, Carlsbad, CA, USA) according to the manufacturer’s instructions. qRT-PCR analysis was performed to detect the level of RNA transcripts. In brief, cDNA was synthesized by M-MLV reverse transcriptase (Invitrogen) from 4 µg of total RNA. Oligo (dT18) RT primer was used for the reverse transcription of mRNA. RT- qPCR was performed on the Bio-rad CFX96 real-time PCR system (Bio-rad, Foster City, CA, USA) using TB Green Fast qPCR Mix (TAKARA, Dalian, China) with the following cycling conditions: 95 °C for 1 min (initial denaturation), followed by 40 cycles of 95 °C for 15 s, 60 °C for 60 s. GAPDH was used for mRNA normalization. Primer sequences are listed in Additional file 2: Table S1.

### Oligonucleotides and constructs

The shRNA specific to hnRNPA2B1 and control shRNAs (shRNA-control) were synthesized by Ribobio (Guangzhou RiboBio Co., Ltd., China) and transfected (100 nM) using Lipofectamine RNAiMAX (Invitrogen, Carlsbad, CA, USA). For BIRC5 isoform overexpression, the human BIRC5-202 and -203 cDNA ORF was synthesized and inserted into the pcDNA3.1 vector (pcDNA-BIRC5-202 or pcDNA-BIRC5-203) by SyngenTech (China). Transfection of the constructs was carried out with Lipofectamine 3000 (Invitrogen) for GC cells according to the manufacturer’s protocols.

### Cell proliferation assay

HGC-27 and MGC-803 cells were incubated in 10 % CCK-8 (DOJINDO, Japan) diluted in normal culture medium at 37 °C until visual color conversion occurred. Proliferation rates were determined at 0, 24, 48, 72 and 96 h after transfection. The absorbance of each well was measured with a microplate reader set at 450 and 630 nm. All experiments were performed in triplicate.

### Apoptosis assay

HGC-27 cells were stained by using Annexin V/PI Cell Apoptosis Kit (DOJINDO, Japan) according to the manufacturer’s recommendations and apoptosis was analyzed by BD Accuri C6 Flow cytometer (BD Biosciences, San Jose, CA, USA).

### Cell migration and invasion assays

For the transwell assays, after 24 h of transfection, 1 × 10^5^ HGC-27 cells in serum-free media were seeded onto the transwell migration chambers (8 μm pore size; Millipore, Switzerland), in which the upper chamber of an insert was coated with Matrigel (Sigma-Aldrich, St. Louis, MO, USA). Media containing 20 % FBS were added to the lower chamber. After 24 h, the non-invading cells were removed with cotton wool. Invasive cells located on the lower surface of the chamber were stained with May–Grunwald–Giemsa stain (Sigma-Aldrich, USA) and counted using a microscope (Olympus, Japan). Experiments were independently repeated three times.

### Spheroid formation assay

HGC-27 cells were suspended into serum-free DMEM culture medium containing 1 % N-2 supplement (Invitrogen), 2 % B-27 supplement (Invitrogen), 1 % antibiotic mixture (Gibco), 20 ng/ml human FGF-2 (R&D Systems, MN, USA), and 100 ng/ml EGF (R&D Systems). Then the cells were plated in 24-well ultra-low attachment plate (Corning, NY, USA) at 500 cells per well. 7–10 days later, plates were analyzed for tumorspheres formation and were quantified using microscope (Olympus) at 40× and 100× magnification, and spheres > 50 μm were counted. Spheroid cells were dissociated with Accutase (Sigma-Aldrich), and monolayer cells were collected with trypsin. For flow cytometry assays, cells were stained with FITC anti-CD44 (eBioscience, CA, USA) according to the manufacturer’s protocol and analyzed by BD Accuri C6 Flow cytometer (BD Biosciences, USA).

### Immunoblotting analysis

Whole-cell lysate or nuclear extract was subjected to immunoblotting analysis using standard methods. Proteins were separated by 10 % SDS-PAGE and transferred onto PVDF membranes (Millipore Corporation, Billerica, MA, USA). Membranes were blocked overnight with 5 % non-fat dried milk for 2 h and incubated with anti-hnRNPA2B1 (1:1000, Abcam) anti-CD44 (1:2000, Cell Signaling Technology) or anti-Sox2 (1:1000; Cell Signaling Technology) antibody overnight at 4 °C. After washing with TBST (10 mM Tris, pH 8.0, 150 mM NaCl, and 0.1 % Tween 20), the membranes were incubated for 2 h at room temperature with goat anti-rabbit or goat anti-mouse antibody (Zsgb-bio, China). All the experiments were repeated at least once with similar results. ImageJ software was used to quantify the Western blot results.

### Immunohistochemistry

Twenty-one paired GC and adjacent normal tissue samples were fixed in 10 % formalin and embedded in paraffin. Tissue Sec. (4 μm) were dewaxed in xylene and rehydrated through a graded series of ethanol washes in distilled water and were then incubated with a 5 % solution of sodium tetraborate. After antigen retrieval and endogenous peroxidase activity blockage, tissue sections were blocked with 5 % bovine serum albumin for 30 min and incubated overnight at 4 °C with anti-hnRNPA2B1 antibody (1:1000, Abcam). EnvisionTM FLEX/HRP (Dako#SM802, Agilent) was used as secondary antibody and chromogenic detection was carried out using EnvisionTM FLEX Substrate Buffer + EnvisionTM FLEX DAB + Chromogen (Dako#SM803, Agilent). Negative controls were obtained by omitting the primary antibodies. Images were captured using an Axio Scan.Z.1 (ZEISS, Germany). The intensity of nuclear staining was scored as 0, 1, 2, 3 and the proportion of cells stained for each intensity was scored as 0-100. The multiplication of the nuclear staining intensity and nuclear staining extent scores was used as the staining score for hnRNPA2B1, which was ranged between 0 and 300.

### RNA immunoprecipitation (RIP)

HGC-27 cells were washed twice with PBS, collected and then the pellet was resuspended in IP lysis buffer (150 mM KCl, 25 mM Tris (pH 7.4), 5 mM EDTA, 0.5 mM DTT, 0.5 % NP40, 1× protease inhibitor, 1 U/µl RNase inhibitor). The lysate was harvested by centrifugation at 12 000 g for 10 min after incubation for 30 min. Antibodies and 40 µl of protein G beads (Invitrogen, USA) were added into the lysate followed by incubation overnight at 4 °C. After washed three times with wash buffer (150 mM KCl, 25 mM Tris (pH 7.4), 5 mM EDTA, 0.5 mM DTT, 0.5 % NP40), co-precipitated RNAs were extracted by Trizol reagent, ethanol-precipitated with glycogen (Invitrogen, USA). The enrichment of RNAs was normalized to IgG.

### Statistics

Each experiment was repeated at least three times. Student’s t-test (two-tailed) was performed for unpaired comparison and three-group data were analyzed using one-way ANOVA. All statistical analyses were performed using Prism 8 software (GraphPad, USA). Statistically significance was set at p < 0.05.

### Data availability

These data were derived from the following resources available in the public domain: cBioPortal and https://www.cbioportal.org/.

## Results

### hnRNPA2B1 is highly expressed in GC and correlates with adverse prognosis

**Fig. 1:**
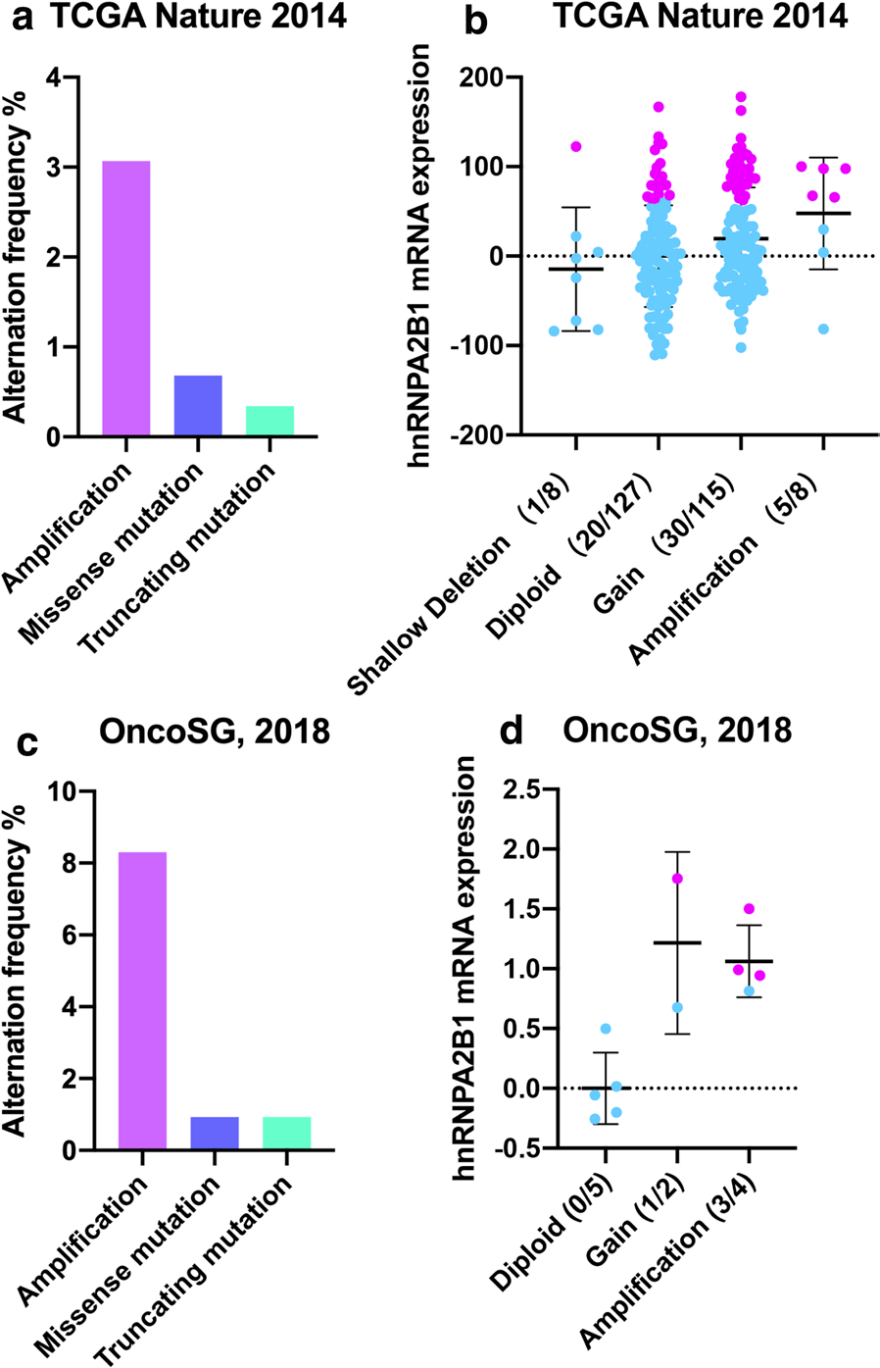
Genetic alternation of hnRNPA2B1 in GC patients. **a**, **b** Gene mutation rates of hnRNPA2B1 in GC patients according to cBioPortal datasets. **c**, **d** Correlation analysis between the copy number status and gene expression in the corresponding cBioPortal datasets

**Fig. 2:**
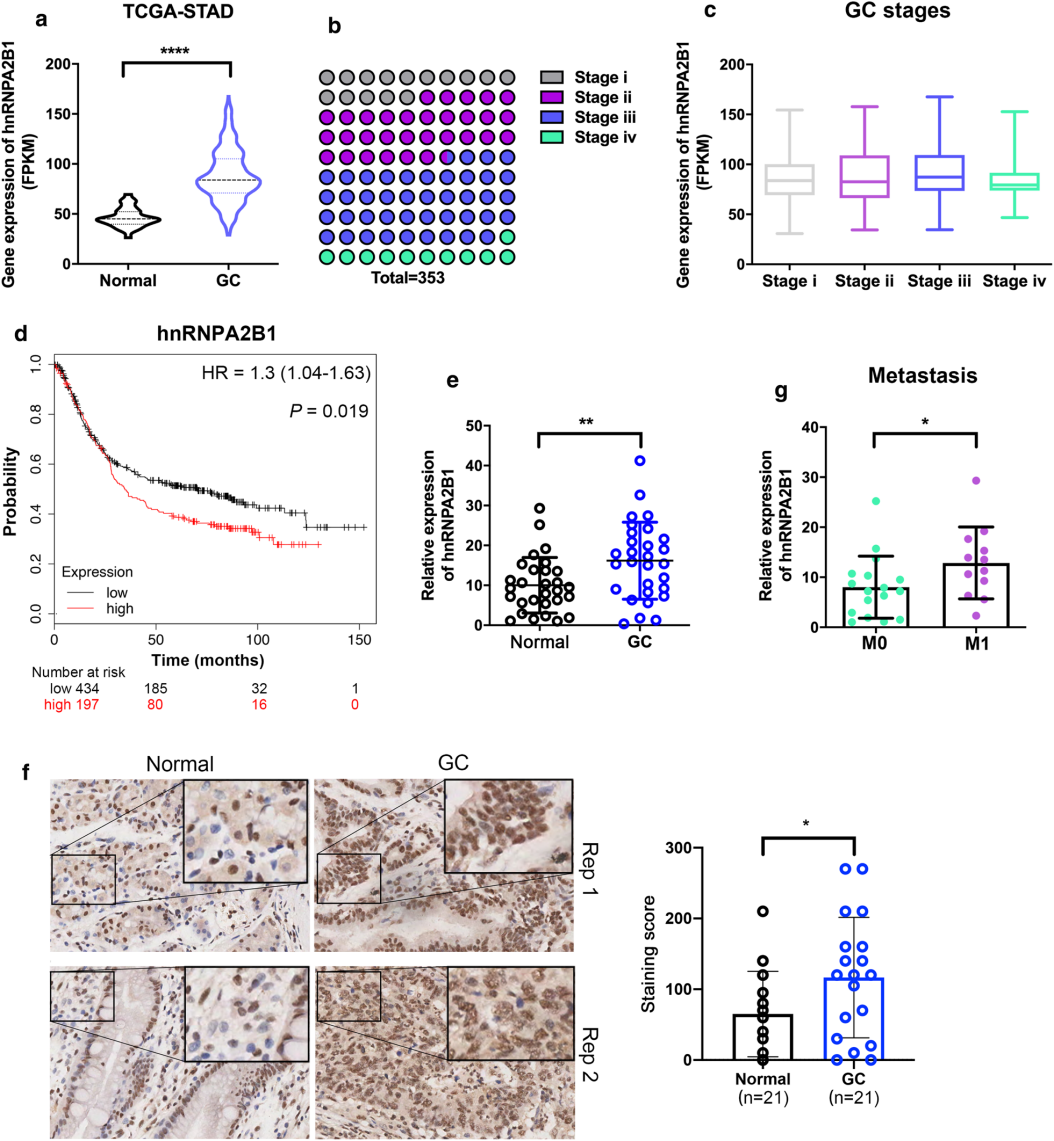
hnRNPA2B1 expression is elevated in GC and correlated with adverse prognosis. **a** The hnRNPA2B1 mRNA expression levels in adjacent non-cancerous tissues and GC tissues from TCGA dataset. **b** The distribution of neoplasm histologic stages in GC patients of TCGA dataset. **c** The hnRNPA2B1 mRNA expression in patients with different stages. **d** Overall survival for GC patients with high (n = 197) and low (n = 434) hnRNPA2B1 expression by Kaplan–Meier curve. **e** qRT-PCR results of hnRNPA2B1 expression in 29 paired primary GC tissues and adjacent non-cancerous tissues. **f** Immunohistochemistry staining of hnRNPA2B1 in primary GC tissues and adjacent non-cancerous tissues (n = 21). Significant increased hnRNPA2B1 protein expression in both nucleus and cytoplasm was observed. The score was obtained by multiplying staining intensity (0–3) and the proportion of pituitary cells stained for each intensity to give a value between 0 and 300. **g** qRT-PCR results of hnRNPA2B1 expression in the above 29 GC tissues with no distant metastases (M0) or with distant metastases (M1). Data are shown as means ± S.D

To examine the role of hnRNPA2B1 in GC development, we initially analyzed its genetic alternation and expression level by using two cohorts of GC dataset in cBioPortal (cBio Cancer Genomics Portal). *hnRNPA2B1* gene was amplified in 3.07 % of TCGA samples and 8.3 % of OncoSG samples (Fig. 1a, b), and positive correlation of the copy number of *hnRNPA2B1* and its mRNA expression in these two cohorts was found (Fig. 1c, d). Further expression analysis in TCGA dataset showed that hnRNPA2B1 was significantly overexpressed in GC tissues when compared with the adjacent non-cancerous tissues (Fig. 2a). To investigate the correlation of dysregulated hnRNPA2B1 expression with GC progression, we analyzed hnRNPA2B1 expression levels with the clinical characteristics of GC patients in this cohort (Fig. 2b). Although no correlation was observed between more aggressive tumor stages and hnRNPA2B1 expression (Fig. 2c), GC patients with high hnRNPA2B1 expression displayed more adverse overall survival by Kaplan–Meier survival analysis (p < 0.001, Fig. 2d).

To validate these results, we performed real-time qRT-PCR analysis in GC samples, and confirmed the ectopic upregulation of hnRNPA2B1 in GC tumors compared with paired adjacent normal tissues (p = 0.0092, Fig. 2e). Immunohistochemistry (IHC) results also demonstrated the elevated hnRNPA2B1 protein expression in GC tissues (Fig. 2f). Intriguingly, high hnRNPA2B1 expression also significantly correlated with metastasis (p = 0.0438) in GC patients but not with other features, including invasion, location, sex and age (Fig. 2 g, Additional file 1: Figure S1).

### hnRNPA2B1 expression in GC cell lines

**Fig. 3:**
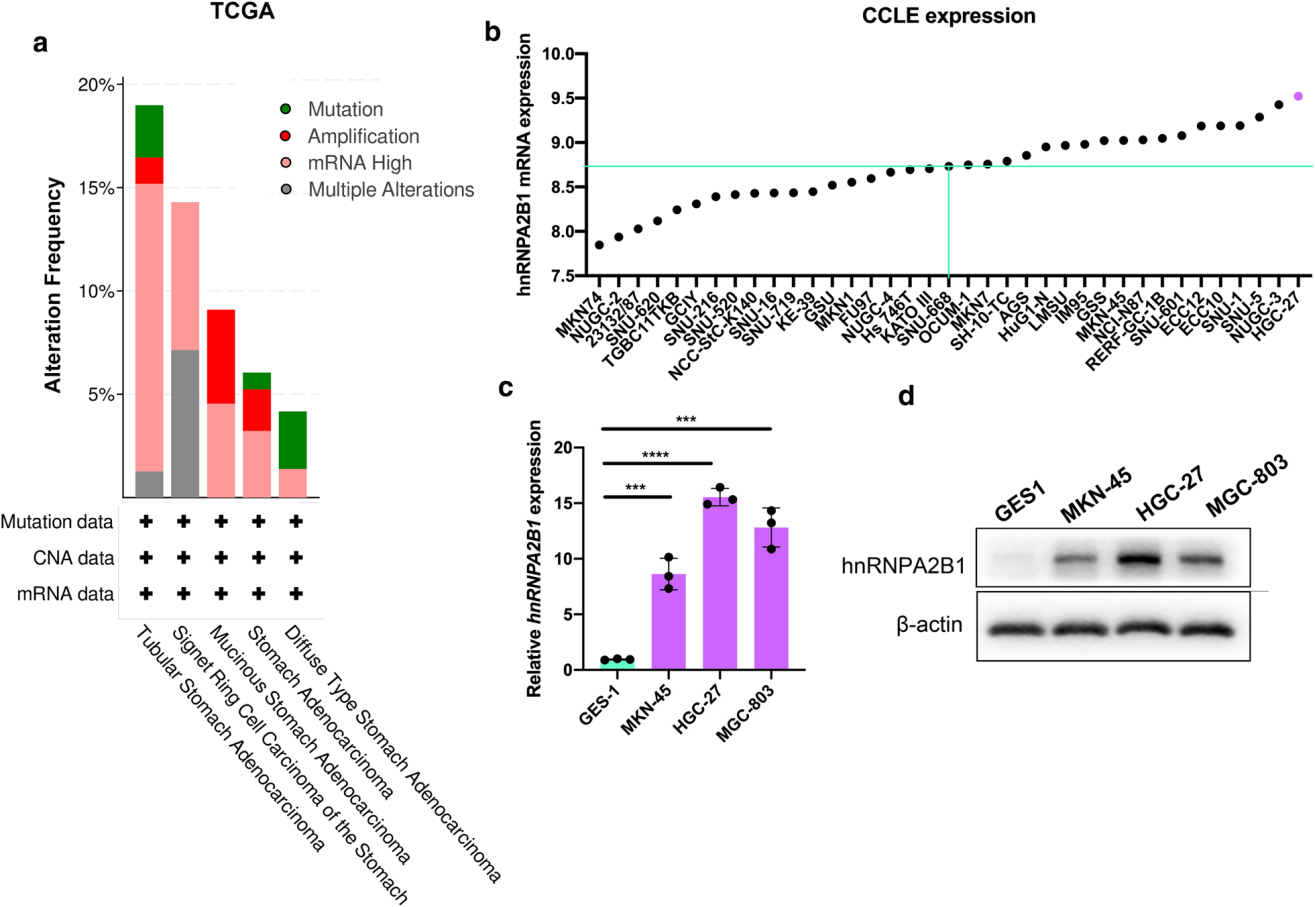
hnRNPA2B1 expression in GC subtypes and GC cell lines. **a** Gene mutation rates of hnRNPA2B1 in GC patients stratified by tumor subtypes according to cBioPortal datasets. **b** hnRNPA2B1 expression in 37 GC cell lines according to CCLE database (https://portals.broadinstitute.org/ccle). **c**, **d** hnRNPA2B1 mRNA (**c**) and protein (**d**) levels in MKN-45, HGC-27 and MGC-803 GC cell lines as compared with the normal gastric epithelial cell line GES1. Data are shown as means ± S.D

As each histological type of GC was identified to have specific molecular characteristics, we assessed the association between hnRNPA2B1 and GC subtypes in TCGA dataset. As shown in Fig. 3a, hnRNPA2B1 overexpression was observed in all types of GC while most *hnRNPA2B1* amplification was found among mucinous stomach adenocarcinoma. We further analyzed hnRNPA2B1 expression in multiple GC cell lines by data mining in the CCLE (Broad Institute Cancer Cell Line Encyclopedia). Among the 37 available cell lines, hnRNPA2B1 was highly expressed in mucinous stomach adenocarcinoma cell line (HGC-27), poorly differentiated adenocarcinoma cell lines (NUGC-3, SNU-5, SNU-3) and small cell gastrointestinal carcinoma cell lines (ECC10 and ECC12) (Fig. 3b). In support of the above results, Western blotting and qRT-PCR confirmed elevated hnRNPA1B1 level in GC cells compared with the GES-1 cells (gastric epithelial mucosa cells) (Fig. 3c, d). Again, the two mucinous stomach adenocarcinoma cell line HGC-27 and MGC-803 showed higher hnRNPA2B1 expression (Fig. 3c, d), which were taken for functional studies.

### hnRNPA2B1 promotes GC cell proliferation and enhances cell metastasis

**Fig. 4:**
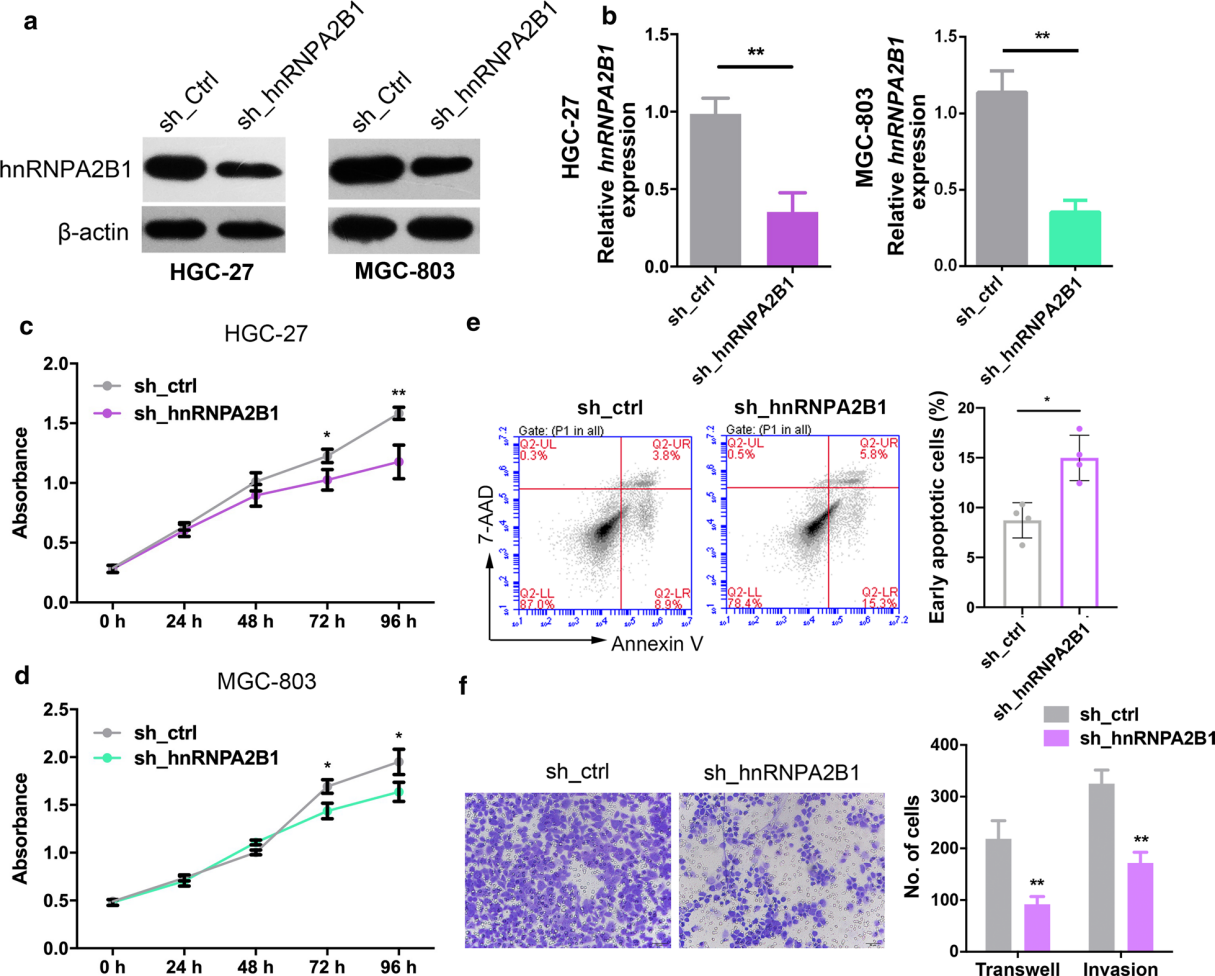
hnRNPA2B1 knockdown suppresses cell proliferation. **a**, **b** Western blotting (**a**) and qRT-PCR analysis (**b**) for hnRNPA2B1 expression in HGC-27 and MGC-803 cells transfected with shRNAs targeting hnRNPA2B1 or a control shRNA. **c**, **d** CCK8 assays were performed to assess cell growth after hnRNPA2B1 was inhibited in HGC-27 (**c**) and MGC-803 (**d**) cells. **e** hnRNPA2B1 knockdown increased cell apoptosis in HGC-27 cells. **f** Knockdown of hnRNPA2B1 decreased the abilities of migration and invasion of HGC-27 cells. Scale bar, 200 μm. Data are shown as means ± S.D. *p < 0.05, **p < 0.01, ***p < 0.001

To investigate the biological functions of hnRNPA2B1 in GC, we transfected HGC-27 and MGC-803 cells with hnRNPA2B1 shRNA or an empty vector and confirmed at both mRNA and protein levels (Fig. 4a, b). CCK8 proliferation assays showed that inhibition of hnRNPA2B1 expression significantly inhibited GC cell proliferation compared with the control group cells (Fig. 4c, d). In spite of cell proliferation block, hnRNPA2B1 shRNA also induced elevated levels of apoptosis, as measured using flow cytometry assays (Fig. 4e). We next investigated the role of hnRNPA2B1 in GC cell movement. Knockdown of hnRNPA2B1 can significantly reduce the number of invasive GC cells passing through Matrigel-coated membrane matrix compared with control (Fig. 4f). The function of hnRNPA2B1 in GC cells was further validated by using another shRNA targeting hnRNPA2B1 (Additional file 1: Figure S2a–e). Therefore, hnRNPA2B1 functioned oncogenic roles in GC by promoting cell proliferation, suppressing cell apoptosis as well as increasing cell metastasis.

### hnRNPA2B1 knockdown reduces cancer stem cell characteristics

**Fig. 5:**
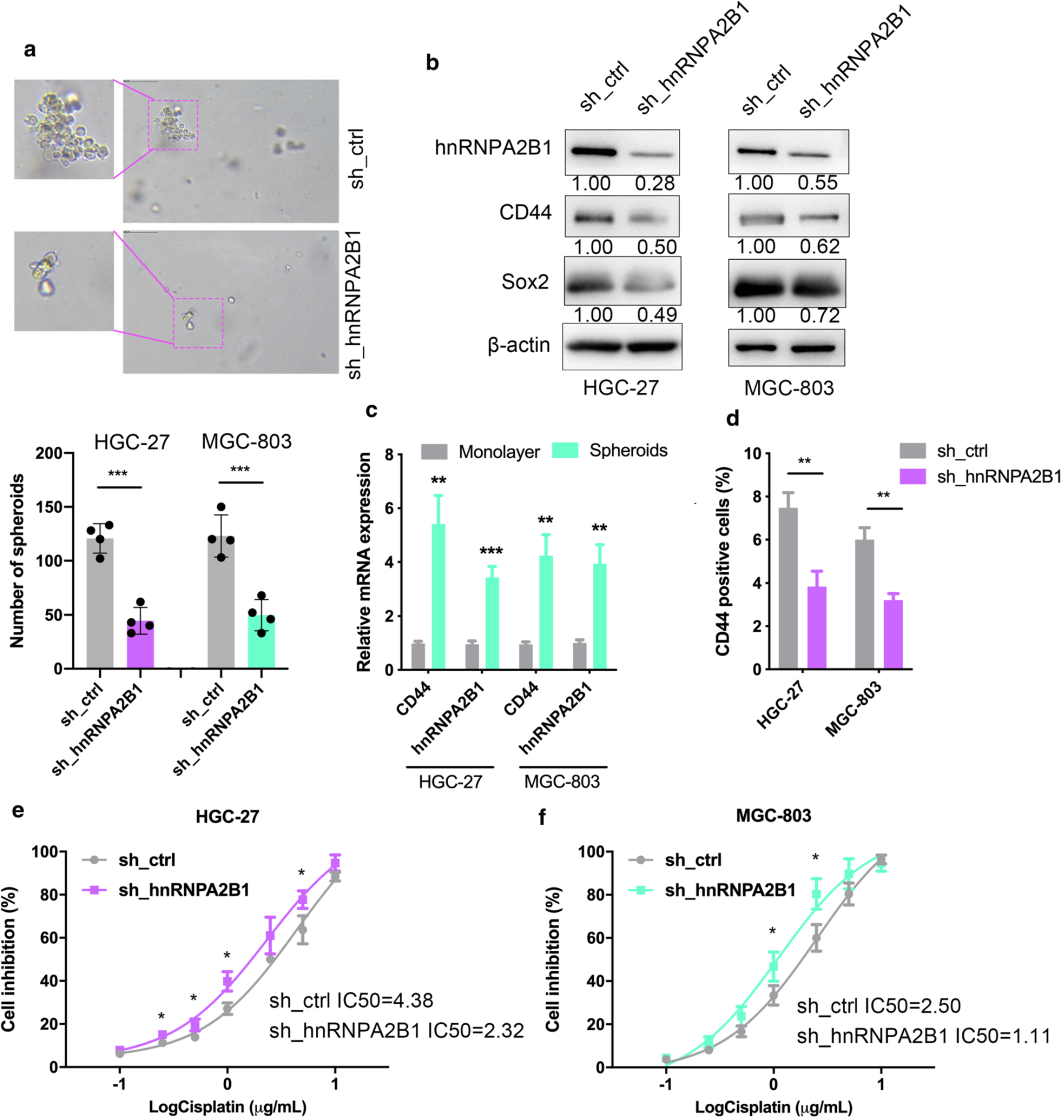
hnRNPA2B1 regulated cancer stem–like cell features of GC. **a** Tumorsphere assays were performed to assess the cancer stemness of HGC-27 and MGC-803 cells. Upper, representative images are shown. Lower, statistical graphs. **b** Western blotting of hnRNPA2B1 and CSC markers, CD44 and Sox2 expression in HGC-27 and MGC-803 cells upon hnRNPA2B1 knockdown. **c** qRT-PCR was performed to evaluate the mRNA expression levels of CD44 and hnRNPA2B1 in monolayer GC cells and tumorspheres obtained from GC cells. **d** FACS analysis of CD44-positive cells by for tumorsphere cells after transfection with control shRNA or hnRNPA2B1 shRNA. The percentage was shown. **e**, **f** The results from HGC-27 cells (**e**) or MGC-803 cells (**f**) transfected with control shRNA or a shRNAs targeting hnRNPA2B1 and treated with cisplatin for 48 h. The IC50 value was determined by Prism 8 software. Data are presented as means ± S.D

Recently, it is recognized that metastases can be initiated by certain subpopulations of cancer stem cells (CSCs). We hence examined tumorsphere formation ability and CD44 (the gastric CSC marker) expression in GC cells following modulation of hnRNPA2B1 levels. The results demonstrated that inhibition of hnRNPA2B1 markedly reduced the tumorsphere formation abilities of GC cells (Fig. 5a, Additional file 1: Figure S2d, f). Western blotting showed that HGC-27 and MGC-803 cells with hnRNPA2B1 knockdown displayed significant decreased expression of both CD44 and self-renewal protein Sox2 (Fig. 5b, Additional file 1: Figure S2g). In tumorspheres (obtained from spheroid formation assays), which were considered to be formed by CSCs, the mRNA expression levels of CD44 and hnRNPA2B1 significantly increased when compared with that in monolayer cells (Fig. 5c). In addition, CD44 expression decreased nearly to one half in GC cells derived tumorspheres with hnRNPA2B1 knockdown, as determined by flow cytometry (Fig. 5d). As CSCs are considered to be responsible for chemoresistance [18], we assessed whether hnRNPA2B1 is also involved in chemoresistance. Upon treatment with cisplatin, HGC-27 cells silencing of hnRNPA2B1 exhibited enhanced sensitivity (Fig. 5e). Similar results were observed in MGC-803 cells (Fig. 5f). Collectively, the above data indicated that hnRNPA2B1 positively contributes to CSC phenotypes of GC cells.

### Co-regulated cell growth and mRNA processing program by hnRNPA2B1 in GC

**Fig. 6:**
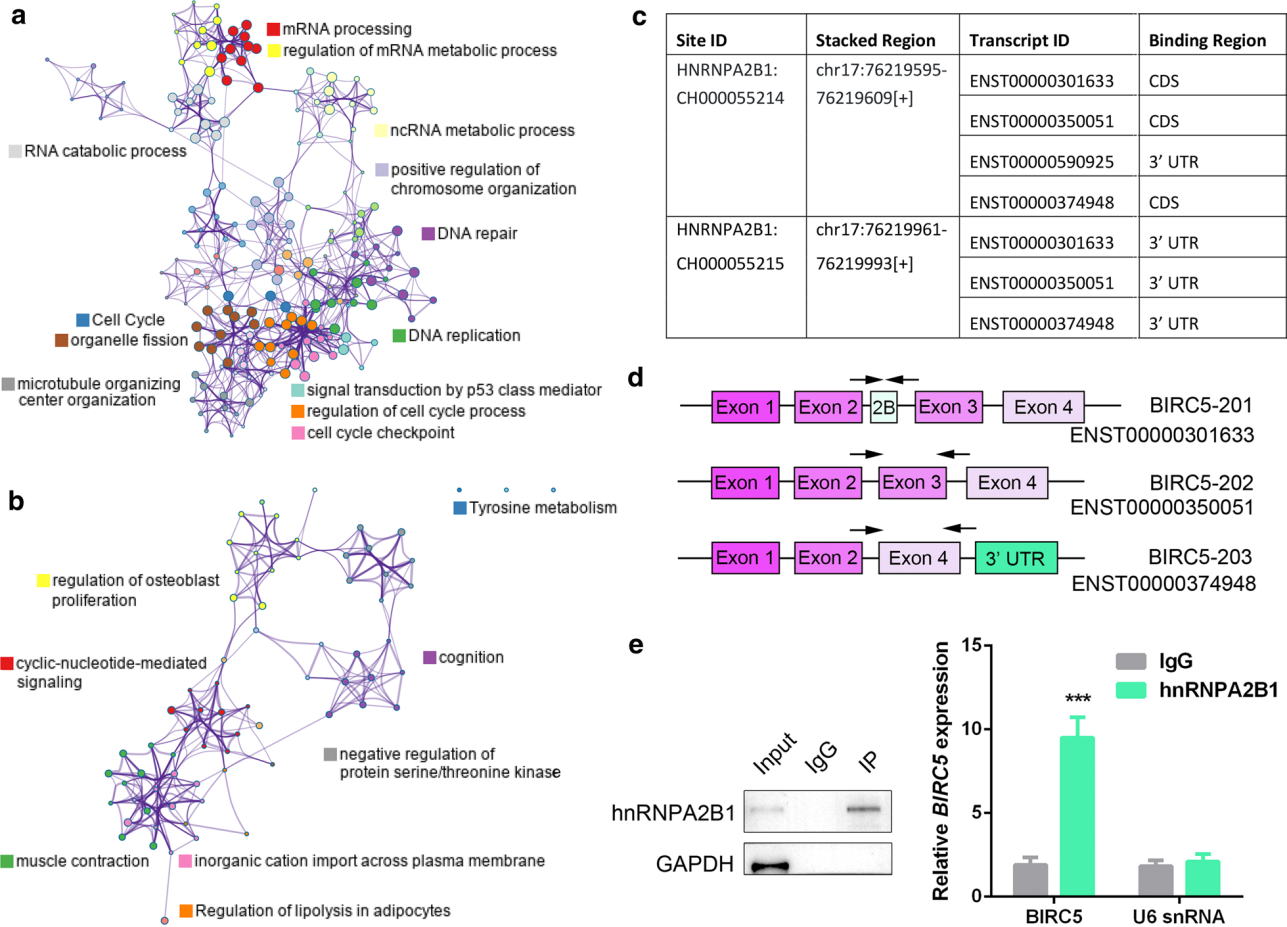
Identification and validation of the hnRNPA2B1 target mRNAs in GC cells. **a** GO enrichment analysis of hnRNPA2B1 positively co-expressed genes (Pearson > 0.6, padj < 0.05) in cBioPortal TCGA dataset. **b** GO enrichment analysis of hnRNPA2B1 negatively co-expressed genes (Pearson < -0.6, padj < 0.05) in cBioPortal TCGA dataset. **c** The hnRNPA2B1 binding sites on BIRC5 mRNA by analyzing ENCORI database. **d** Schematic representation of the exons encoding three protein coding isoforms of BIRC5 in Ensembl. Arrows indicated the sites for qRT-PCR primers. **e** RIP-PCR analysis for the interaction of hnRNPA2B1 protein and BIRC5 mRNA. Data are presented as means ± S.D

To explore the mechanism underlying elevated hnRNPA2B1 expression in GC, we first screened the genes co-expressed with hnRNPA2B1 in TCGA dataset. Among them, many known oncogenes involved in cell proliferation and metastasis, such as BRCA1, CCNA2, CCNB1, CDC25A, PCNA, DDX39B etc. were positively correlated with hnRNPA2B1 expression (Additional file 3: Table S2). Particularly, numerous genes encoding proteins associated with the spliceosome complex were also found. On the contrary, a few known tumor suppressor genes, such as RERG and SRPX showed negative correlation with hnRNPA2B1 expression (Additional file 4: Table S3). Further, the highly hnRNPA2B1 co-expressed genes (|Pearson| > 0.6, padj < 0.05) were subjected to gene ontology (GO) analysis by Metascape [19]. The top 6 terms enriched by positive-coexpressed genes were mRNA processing, cell cycle, DNA replication, DNA repair, regulation of cell cycle process and regulation of mRNA metabolic process (Fig. 6a, Additional file 1: Figure S3a). In contrast, negative-coexpressed genes enriched terms including cyclic-nucleotide-mediated signaling, tyrosine metabolism, regulation of lipolysis in adipocytes, positive regulation of extrinsic apoptotic signaling pathway (Fig. 6b, Additional file 1: Figure S3b). The above results place hnRNPA2B1 in an extended gene regulatory network that is essential for GC development and suggest the importance of hnRNPA2B1 in modulation of mRNA splicing.

### hnRNPA2B1 regulates the alternative splicing of BIRC5

Given the co-expression of core splicing factors with hnRNPA2B1 and previous reports identifying alternative splicing of exons mediated by hnRNPA2B1 [20, 21], we hypothesized that loss of hnRNPA2B1 might disrupt splicing and expression of mRNAs preferentially required for GC development. On this ground, we firstly analyzed hnRNPA2B1 CLIP-seq data of HepG2 tumor cells in ENCORI database [22] to investigate its directly interacted transcripts. Two hnRNPA2B1 binding sites were identified at BIRC5 mRNA, in which the first site was located at the last exon (exon 4) of the protein coding isoforms (Fig. 6c, d). To verify the interaction, RIP (RNA immunoprecipitation)-qPCR was performed in HGC-27 cells and the results showed that BIRC5 mRNA was enriched by hnRNPA2B1 (Fig. 6e, Additional file 1: Figure S4). These data suggest that BIRC5 is a physical target of hnRNPA2B1 in GC cells.

**Fig. 7:**
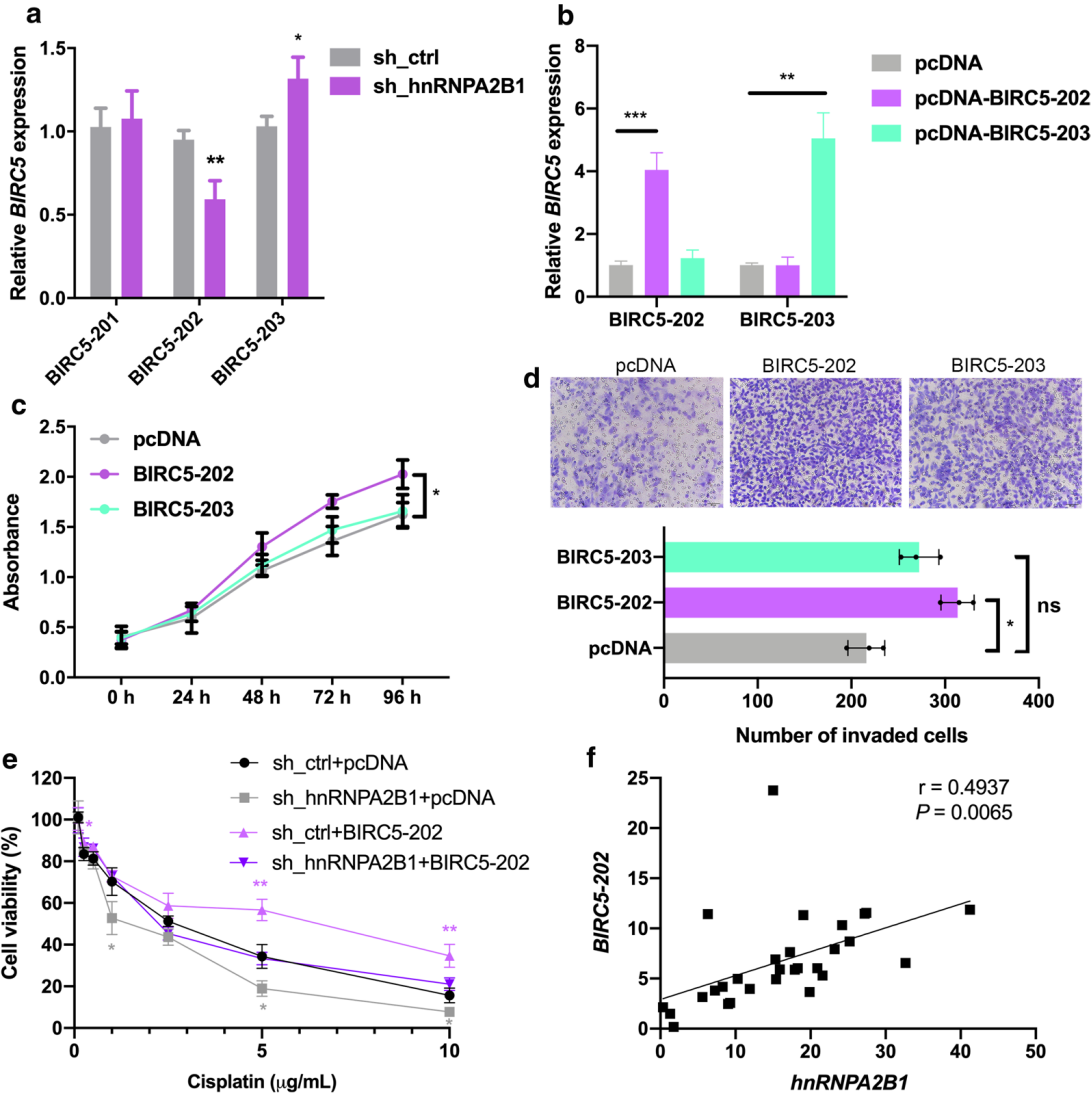
Overexpression of BIRC5-202 partially rescues the chemosensitivity of hnRNPA2B1-knockdown GC cells. **a** Expression levels of BIRC5-201, -202 and -203 mRNA in HGC-27 cells after transfection with control shRNA or hnRNPA2B1 shRNA. **b** pcDNA vector, pcDNA-BIRC5-202 or pcDNA-BIRC5-203 were transfected into HGC-27 cells and the expression of each isoforms were determined by qRT-PCR. **c** CCK8 assays were performed to determine cell growth in HGC-27 cells as treated in **b**. **d** Cell invasion ability of HGC-27 cells as treated in **b**. **e** BIRC5-202 was overexpressed in hnRNPA2B1 shRNA or shRNA control transfected HGC-27 cells. Cell viability was examined after cisplatin treatment for 48 h. **f** Correlation of BIRC5-202 and hnRNPA2B1 mRNA expression, as determined by qRT-PCR, in 29 GC samples. Data are presented as means ± S.D

BIRC5 (survivin) mRNA expression levels were associated with poor prognosis, and inhibition of BIRC5 expression is connected to diverse aspects of cancer progression, including increased apoptosis, impaired cell migration and decreased cisplatin-resistance in GC [23, 24]. To investigate hnRNPA2B1 regulating on BIRC5 mRNA, we examined the expression of the protein-coding BIRC5 isoforms, namely BIRC5-201, -202, and -203, with specific primer pairs (Fig. 6d). hnRNPA2B1 silencing by shRNA in HGC-27 cells resulted in different changes of these isoforms, significantly reducing BIRC5-202 but increasing BIRC5-203 expression while unaffecting BIRC5-201 expression (Fig. 7a). This result, in combination with the hnRNPA2B1 binding site at exon4, supporting that hnRNPA2B1 promotes the splicing of BIRC5-203 at the expense of BIRC5-202.

### BIRC5-202 plays an oncogenic role in GC

As different BIRC5 isoforms showed distinct links with chemotherapy responses and the function of each isoforms in GC is not fully understood [25], we analyzed the cellular phenotypes upon BIRC5-202 or -203 overexpression in GC by transfection of pcDNA-BIRC5-202 or -203 in HGC-27 cells (Fig. 7b). HGC-27 was used since hnRNPA2B1 showed highest expression in this cell line all among CCLE GC cell lines (Fig. 3b). As expected, BIRC5-202 overexpression promoted cell proliferation and invasion, however, BIRC5-203 overexpression showed no effect on cell proliferation or invasion (Fig. 7c, d). The above results suggested that BIRC5-202, but not BIRC5-203, promotes malignant capacity in GC. To further illustrate whether hnRNPA2B1 modulates BIRC5-202 production to impact chemotherapy responses of GC cells, a rescue assay was performed by overexpression BIRC5-202 in hnRNPA2B1-knockdown HGC-27 cells. BIRC5-202 overexpression alone led to increases in cell viability upon cisplatin treatment, consistently, cisplatin-resistance reduced by hnRNPA2B1 knockdown were re-established after expression of BIRC5-202 was restored (Fig. 7e). Finally, we examined the relationship between hnRNPA2B1 and BIRC5-202 expression in GC tissues, and a positive correlation between these two mRNA was found (Fig. 7f). Taken together, our data confirmed that BIRC5-202 is a critical downstream target of hnRNPA2B1 to relay the oncogenic signal in GC.

## Discussion

Increasing evidence has revealed that aberrant splicing program is responsible for human neoplasms, including several digestive tract malignancy [26]. Efficient pre-mRNA splicing required both cis-elements and trans-acting regulators, and their dysregulations induce aberrantly spliced molecules, resulting in aberrant expression profiles. Several studies have demonstrated that splicing factors are frequently mutated or dysregulated in GC and pre-mRNA splicing is frequently deregulated in the disease, which may also correlate with prognostic value [27, 28]. In addition, we and others have identified the molecular pathways that regulates stem cell marker CD44 variant isoform expression in GC [29, 30]. Thus targeting alternative splicing showed great therapeutic potential for the treatment of GC.

Here, we focused on a splicing factor, hnRNPA2B1, due to its extensive oncogenic function among solid tumors. hnRNPA2B1 overexpression was found in GC tissues from two cohorts of GC datasets, and higher hnRNPA2B1 expression was correlated with gene amplification. More importantly, patients with high hnRNPA2B1 expression showed worse prognosis, suggesting the expression level of hnRNPA2B1 may affect the patient’s response to chemotherapy. We analyzed the function of hnRNPA2B1 in two mucinous GC cell lines, HGC-27 and MGC-803, which displayed highest hnRNPA2B1 expression. Concurrent with its aberrant expression, hnRNPA2B1 promoted cell proliferation, inhibited cell apoptosis and increased cell metastasis. We provided further evidence showing that hnRNPA2B1 promoted the acquirement of CSC properties of GC cells. With hnRNPA2B1 shRNA transfection, GC cells showed a decrease in tumorsphere formation ability and stem cell marker expression. Moreover, hnRNPA2B1 shRNA transfection resulted in an reduce in the IC50 of cisplatin GC cells. Furthermore, inhibition of endogenous hnRNPA2B1 can increase the drug sensitivity of pancreatic and ovarian cancer cells, which is consistent with our findings [31, 32].

As an RNA binding protein, hnRNPA2B1 affects RNA fates in multiple manners and thus regulates oncogenic signaling pathways. It is reported to directly bind and regulate mRNA stability in ovarian cancer cells and hepatocytes [9, 33]. In addition, hnRNPA2B1 can interact with phosphorylated KRAS protein, functioning as a regulator of KRAS-dependent tumorigenesis through the critical pancreatic ductal adenocarcinoma signaling pathway PI3K/AKT [10]. In this work, we illustrated that hnRNPA2B1 can bind to BIRC5 pre-mRNA, thus increasing the production of oncogenic BIRC5-202 isoform. Among the three protein-coding isoforms of BIRC5, only BIRC5-202 played the oncogenic function in GC cells by promoting cell proliferation and metastasis. Indeed, overexpression of the BIRC5-202 transcript partly rescued the decrease in cisplatin resistance induced by downregulation of hnRNPA2B1.

The 142 amino acids (aa) long isoform BIRC5-202 was first recognized and intensively studied [34]. Although recurrent mutations in BIRC5 gene is rarely detected, overexpression of BIRC5-202 was prevalent among multiple tumors and inhibits several pathways modulating apoptosis [35]. Hence efforts have been payed to develop BIRC5 inhibitors, such as antisense oligonucleotides, siRNA, dominant-negative mutants, peptidomimetic molecules and other small inhibitory molecules [35, 36]. In GC, higher BIRC5 expression levels were also associated with poor prognosis including decreased survival and lymph node metastasis [37, 38]. In line with our results, inhibition of BIRC5-202 expression is connected to diverse aspects of cancer progression, including increased apoptosis, impaired cell migration and decreased cisplatin-resistance in GC [23, 24]. Therefore, targeting the hnRNPA2B1-BIRC5-202 regulatory pathway could be the adjuvant for traditional chemotherapy.

**Fig. 8:**
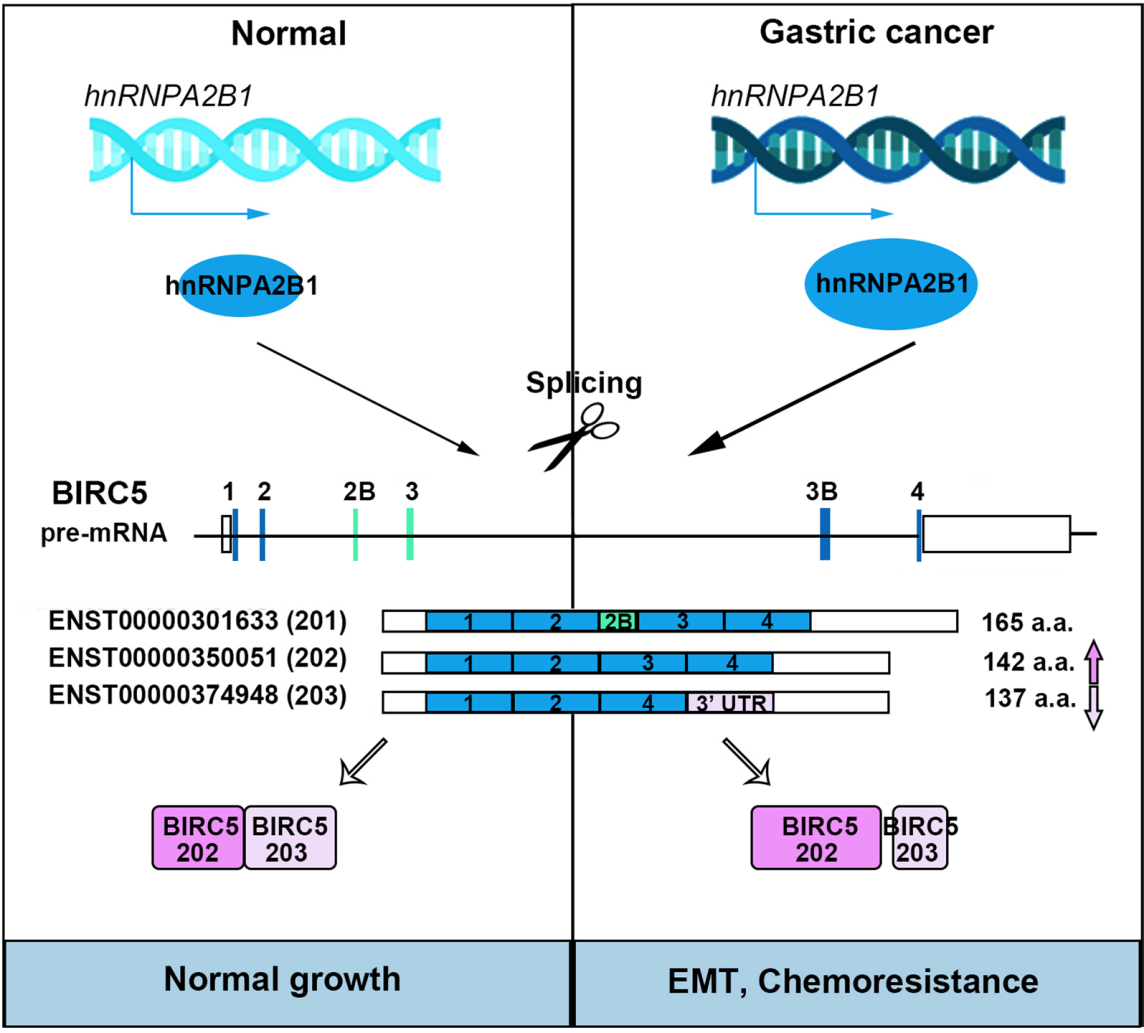
Proposed model underlying the roles of hnRNPA2B1-mediated BIRC5 alternative splicing in GC

Generally, our data proposed the critical function of hnRNPA2B1 in regulating BIRC5 splicing, which is part of molecular control to promote the proliferation and chemoresistance of GC (Fig. 8). In particular, the combination of standard chemotherapy and hnRNPA2B1-BIRC5 inhibitors will more effectively eliminate tumor stem cells and other tumor cells, thereby reducing the incidence of drug resistance.

## Conclusions

hnRNPA2B1 promotes gastric cancer development and chemotherapy resistance partially through increasing the expression of BIRC5-202 transcript, which provide new treating opportunity for chemo-resistant patients.

## Supplementary Information

**Additional file 1.** Supplementary figures.

**Additional file 2: Table S1.** Primer sequences for splice variant expression.

**Additional file 3.** Genes negatively correlated with hnRNPA2B1 expression.

**Additional file 4.** Genes positively correlated with hnRNPA2B1 expression.

## Data Availability

Not applicable.
