# Supplementary material for: hnRNPA2B1 regulates the alternative splicing of BIRC5 to promote gastric cancer progression
Source: Cancer Cell Int. 2021 May 27;21:281. doi: 10.1186/s12935-021-01968-y (PMC8161968; doi:10.1186/s12935-021-01968-y)
Supplement: Supplementary file 1 — Additional file 1. Supplementary figures. [file 12935_2021_1968_MOESM1_ESM.docx]

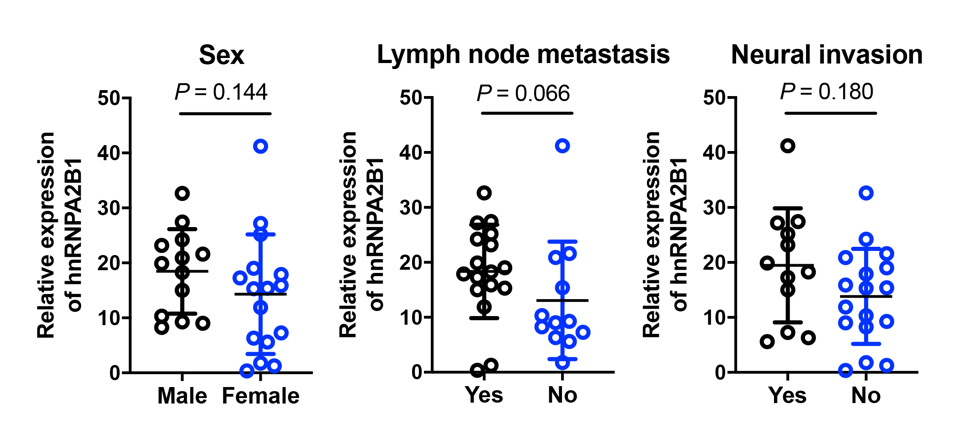


Figure S1. qRT-PCR results of hnRNPA2B1 expression in 29 primary GC tissues that was stratified by sex, location (lymph node metastasis) or neural invasion.


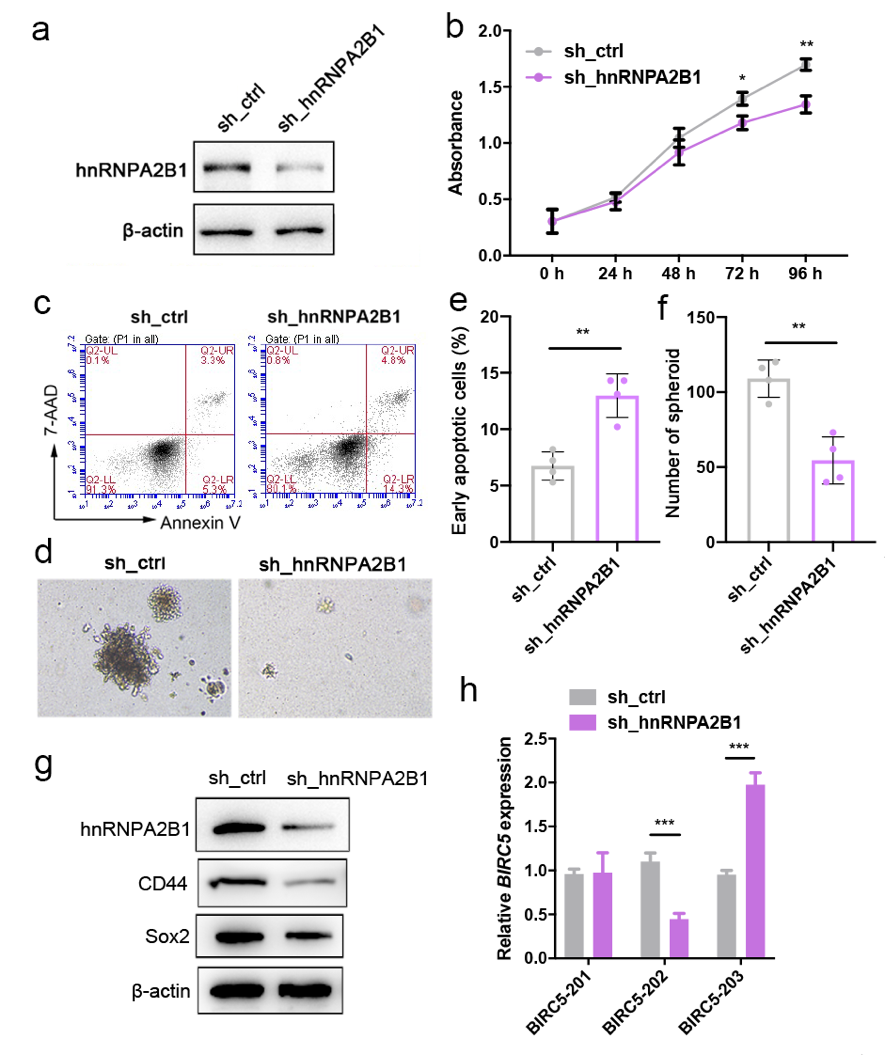


Figure S2. Validating the function of hnRNPA2B1 in HGC-27 cells using another shRNA. (a) Western blotting for hnRNPA2B1 expression in HGC-27 cells transfected with shRNA targeting hnRNPA2B1 or a control shRNA. (b) CCK8 assays were performed to assess cell growth after hnRNPA2B1 was inhibited in HGC-27 cells. (c & e) hnRNPA2B1 knockdown increased cell apoptosis in HGC-27 cells. (d & f) Tumorsphere assays were performed to assess the cancer stemness of HGC-27 cells. Data are shown as means ± S.D. (g) Western blotting of hnRNPA2B1 and CSC markers, CD44 and Sox2 expression in HGC-27 cells upon hnRNPA2B1 knockdown. (h) Expression levels of BIRC5-201, -202 and -203 mRNA in HGC-27 cells after transfection with control shRNA or hnRNPA2B1 shRNA. *P < 0.05, **P < 0.01.


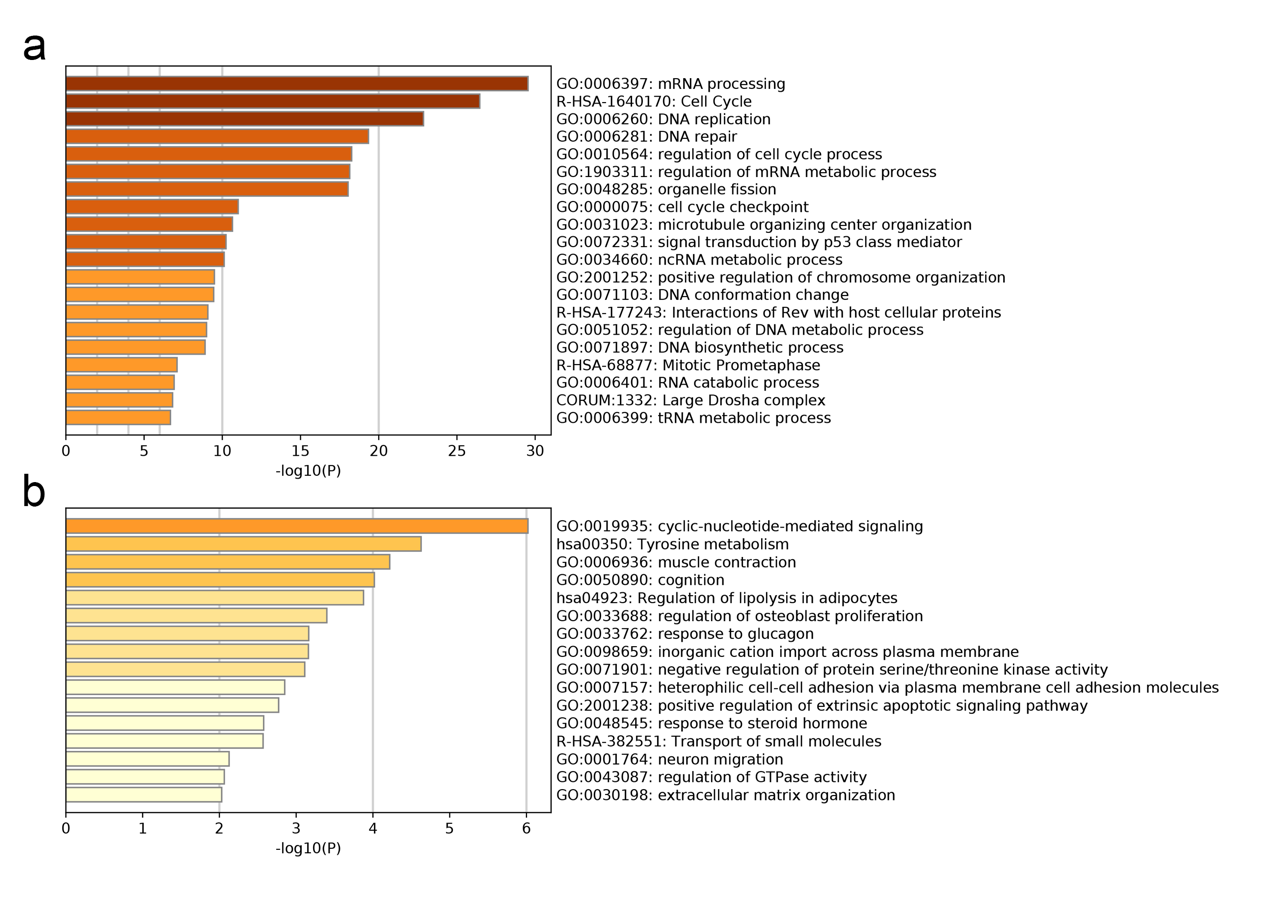


Figure S3. Results of GO term enrichment for genes positively co-expressed (A) and negatively co-expressed (B) with hnRNPA2B1 in GC patients.


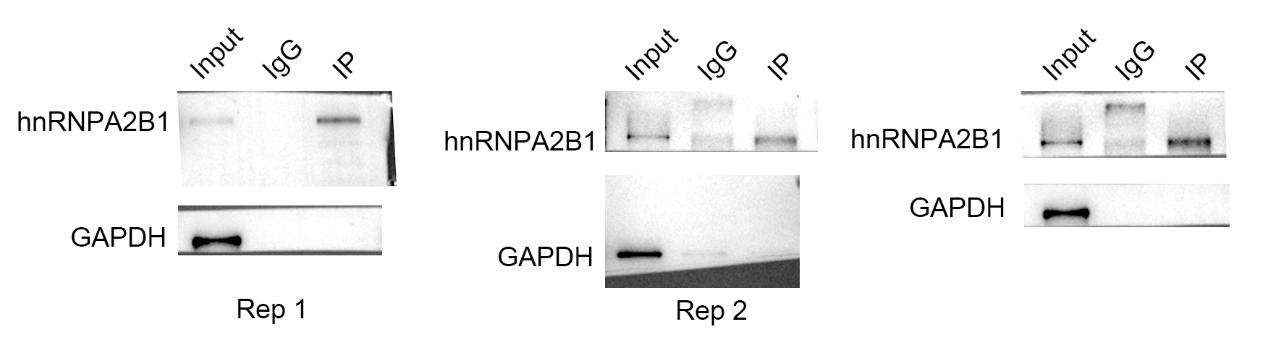


Figure S4. Original pictures of hnRNPA2B1 RIP-PCR as identified by Western blot. The results of triplicates were shown.
