# Supplementary material for: hnRNPA2B1 regulates the alternative splicing of BIRC5 to promote gastric cancer progression
Source: Cancer Cell Int. 2021 May 27;21:281. doi: 10.1186/s12935-021-01968-y (PMC8161968; doi:10.1186/s12935-021-01968-y)
Supplement: Supplementary file 2 — Additional file 2: Table S1. Primer sequences for splice variant expression. [file 12935_2021_1968_MOESM2_ESM.docx]

Table S1: Primer sequences for splice variant expression.

| Gene | Sequence (5'-3') |
| --- | --- |
| hnRNPA2B1 | F – CAGCGGCAGTTCTCACTACA |
|  | R - ATCCCTCATTACCACACAGTCT |
| BIRC5-201 | F - GATGACGACCCCATTGG |
|  | R - TTATGTTCCTCTCTCGTGATCC |
| BIRC5-202 | F - TGACGACCCCATAGAGGAAC |
|  | R - TCCTTTGCATTTTGTTCTTGG |
| BIRC5-203 | F - GACGACCCCATGCAAAG |
|  | R - GTGGCACCAGGGAATAAAC |
| CD44 | F - CCAGAAGGAACAGTGGTTTGGC |
|  | R - ACTGTCCTCTGGGCTTGGTGTT |
| GAPDH | F - CTTTGGTATCGTGGAAGGACTC |
|  | R - CAGTAGAGGCAGGGATGATGTT |
